# Mucositis with targetoid lesions in an adult

**DOI:** 10.1016/j.jdcr.2024.08.022

**Published:** 2024-09-02

**Authors:** Daniel R. Antohi, Tian Zhu, Carson Kirkpatrick, Jose Jaller, Michelle Toker, Benedict Wu

**Affiliations:** a Division of Dermatology, Montefiore Medical Center, Albert Einstein College of Medicine, Bronx, New York; b Department of Pathology, Montefiore Medical Center, Albert Einstein College of Medicine, Bronx, New York

**Keywords:** malar rash, Rowell syndrome, Stevens-Johnson syndrome, systemic lupus erythematosus

## Case presentation

**Fig 1:**
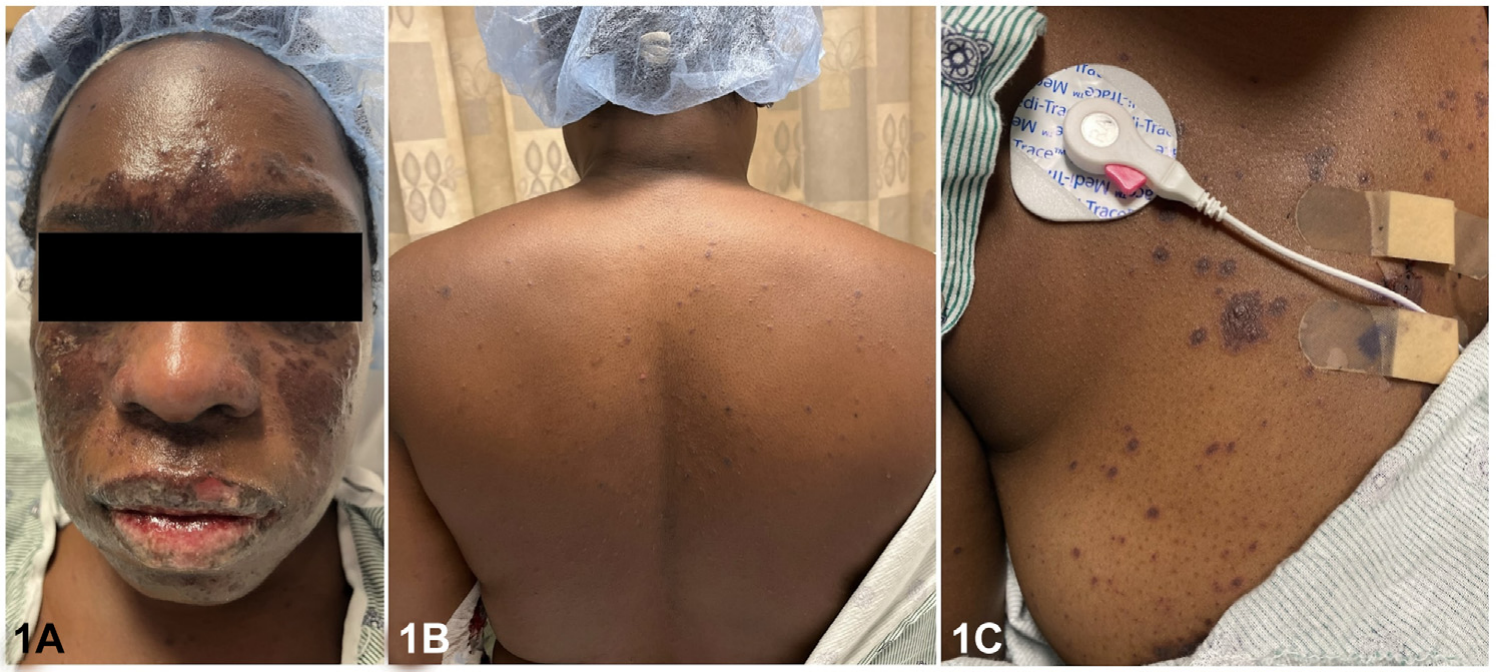

A 34-year-old woman without an autoimmune history was started on lamotrigine 10 days ago for unprovoked seizures. She presents with a 2-day history of Nikolsky-negative violaceous papules and plaques of the malar region (Fig 1, *A*). There were a few macular and atypical targetoid lesions on the trunk (Fig 1, *B* and *C*). The patient had extensive lip and oral erosions with positive antinuclear antibodies (ANA) (titer of 1:160, speckled pattern) and anti-Sjogren's-syndrome-related antigen A (anti-SSA/Ro) antibodies. Extensive testing for viral and bacterial infections was negative. Lesional skin biopsy revealed pauci-inflammatory interface dermatitis with extensive keratinocyte necrosis with a negative direct immunofluorescence study.

**Question 1: What is the most likely diagnosis based on the clinical presentation, laboratory studies, and histopathology?**

A. Stevens-Johnson syndrome (SJS)
B. Rowell syndrome
C. Fixed drug eruption
D. Reactive and infectious mucocutaneous eruption (RIME)
E. Bullous lupus

**Answers:**

A. Stevens-Johnson syndrome (SJS) – Incorrect. SJS would present with dusky targetoid lesions that coalesce into larger patches and plaques without well-demarcated areas of normal intervening skin. Nikolsky-positive lesions and negative ANA would be expected.^1^
B. Rowell syndrome – Correct. Rowell syndrome is characterized by well-defined dusky targetoid lesions with normal intervening skin; lesions can progress to form flaccid bullae with epidermal detachment. This, along with Ro and ANA positivity, strongly favors this diagnosis.^2^
C. Fixed drug eruption – Incorrect. A fixed drug eruption may present as erythematous patches or plaques with overlying vesicles or bullae that occur in the same location on the body after repeated ingestion of a medication. Patients often have a history of a similar reaction, and the most common offending medications are antibiotics and nonsteroidal anti-inflammatory drugs.^3^
D. Reactive and infectious mucocutaneous eruption (RIME) – Incorrect. RIME presents with severe mucosal lesions with less cutaneous involvement.^4^ RIME is a reasonable consideration; however, extensive infectious work-up, which included herpes, Epstein-Barr virus, mycoplasma, and enterovirus, were negative.
E. Bullous lupus – Incorrect. While bullous lupus occurs in patients with positive ANA, the negative direct immunofluorescence and absence of tense bullae is not compatible with bullous lupus.

**Question 2: Patients with this diagnosis must have which of the following?**

A. Centromere autoantibody
B. Rheumatoid factor
C. Ro autoantibody
D. Presence of chilblains
E. Positive ANA with speckled pattern

**Answers:**

A. Centromere autoantibody – Incorrect. This is not part of the Rowell syndrome diagnostic criteria and is more commonly associated with autoimmune diseases such as Sjögren’s syndrome, limited cutaneous systemic sclerosis, and primary biliary cholangitis.
B. Rheumatoid factor – Incorrect. While this is part of the Rowell syndrome criteria, it is a minor criterion and is not a requirement for diagnosis.^2^
C. Ro autoantibody – Incorrect. While this is part of the Rowell syndrome criteria, it is a minor criterion and is not a requirement for diagnosis.^2^
D. Presence of chilblains – Incorrect. While this is part of the Rowell syndrome criteria, it is a minor criterion and is not a requirement for diagnosis.^2^
E. Positive ANA with speckled pattern – Correct. This is part of the Rowell syndrome diagnostic major criteria, which must be fulfilled. The other major criteria include erythema multiforme-like lesions and a diagnosis of systemic lupus erythematosus (SLE), discoid lupus erythematosus, or subacute cutaneous lupus erythematosus (SCLE).^2^

**Question 3: Which of the following findings favored Rowell syndrome over SJS?**

A. Presence of targetoid lesions
B. Mucosal erosions and mucositis
C. Histologic findings show numerous necrotic keratinocytes
D. Prominent malar eruption
E. ANA negativity

**Answers:**

A. Presence of targetoid lesions – Incorrect. This clinical presentation is common in both conditions and would not necessarily favor Rowell syndrome over SJS.
B. Mucosal erosions and mucositis – Incorrect. This clinical presentation is common in both conditions, but especially in SJS. This finding would not favor Rowell syndrome over SJS.
C. Histologic findings show numerous necrotic keratinocytes – Incorrect. This clinical presentation may be seen in both conditions and would not necessarily favor Rowell syndrome over SJS.
D. Prominent malar eruption – Correct. This is consistent with Rowell syndrome and is not typical in SJS.^2^ There are four Rowell syndrome diagnostic criteria. Zeitouni et al require the patient to have SLE, SCLE, or discoid lupus erythematosus. The Torchia et al criteria include those with chronic cutaneous lupus erythematosus and its variants. The Torchia et al criteria accounts for skin-limited lupus variants only because some view Rowell syndrome as a variant of SCLE and acute cutaneous lupus erythematosus (ACLE). In this case, the patient presented with a prominent malar eruption, which was compatible with ACLE. This raised the possibility of underlying SLE since ACLE is most closely associated with SLE. Further evaluation by rheumatology confirmed SLE in this patient based on fever, malar rash, and persistently elevated urine total protein with creatinine ratio. In patients with suspected Rowell syndrome, a thorough workup and review of the classification criteria are necessary since the diagnosis of Rowell syndrome as a distinct entity remains controversial.
E. ANA negativity – Incorrect. Rowell syndrome requires ANA positivity, and thus, its absence would not favor Rowell syndrome over SJS.^2^

## Conflicts of interest

None declared.
